# Supplementary material for: Novel Driver Strength Index highlights important cancer genes in TCGA PanCanAtlas patients
Source: PeerJ. 2022 Aug 11;10:e13860. doi: 10.7717/peerj.13860 (PMC9375969; doi:10.7717/peerj.13860)
Supplement: Supplemental Information 2 [file peerj-10-13860-s002.zip › COHORTS/genes plots/detailed/2021_8_16_14_9_distribution_events_detailed_females_32.pdf]

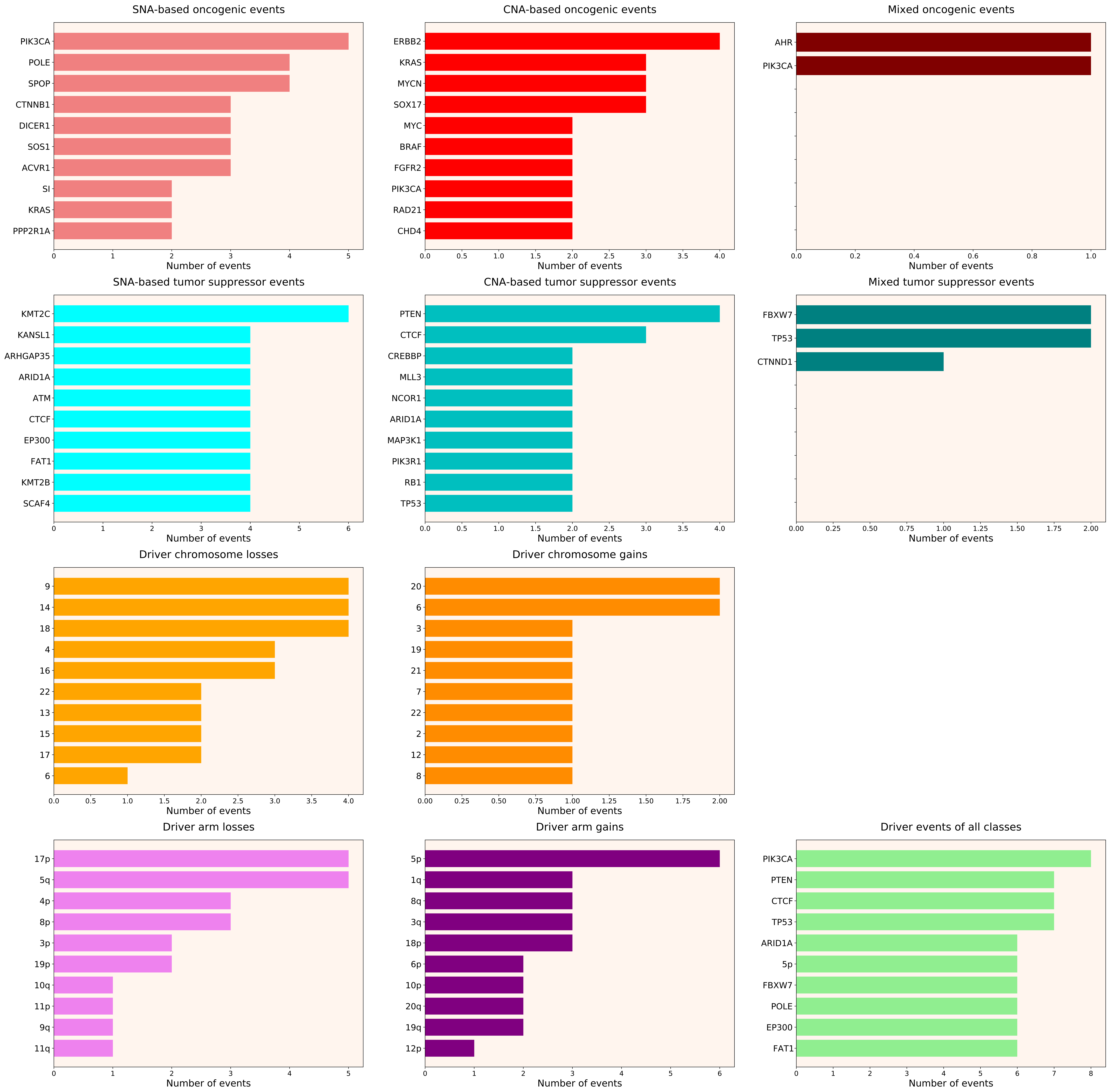

SNA-based oncogenic events
PIK3CA
POLE
SPOP
CTNNB1
DICER1
SOS1
ACVR1
SI
KRAS
PPP2R1A
Number of events
CNA-based oncogenic events
ERBB2
KRAS
MYCN
SOX17
MYC
BRAF
FGFR2
PIK3CA
RAD21
CHD4
Number of events
Mixed oncogenic events
AHR
PIK3CA
Number of events
SNA-based tumor suppressor events
KMT2C
KANSL1
ARHGAP35
ARID1A
ATM
CTCF
EP300
FAT1
KMT2B
SCAF4
Number of events
CNA-based tumor suppressor events
PTEN
CTCF
CREBBP
MLL3
NCOR1
ARID1A
MAP3K1
PIK3R1
RB1
TP53
Number of events
Mixed tumor suppressor events
FBXW7
TP53
CTNND1
Number of events
Driver chromosome losses
9
14
18
4
16
22
13
15
17
6
Number of events
Driver chromosome gains
20
6
3
19
21
7
22
2
12
8
Number of events
Driver arm losses
17p
5q
4p
8p
3p
19p
10q
11p
9q
11q
Number of events
Driver arm gains
5p
1q
8q
3q
18p
6p
10p
20q
19q
12p
Number of events
Driver events of all classes
PIK3CA
PTEN
CTCF
TP53
ARID1A
5p
FBXW7
POLE
EP300
FAT1
Number of events
